# Proteomic analysis of *Rickettsia akari* proposes a 44 kDa-OMP as a potential biomarker for Rickettsialpox diagnosis

**DOI:** 10.1186/s12866-020-01877-6

**Published:** 2020-07-08

**Authors:** František Csicsay, Gabriela Flores-Ramirez, Fernando Zuñiga-Navarrete, Mária Bartošová, Alena Fučíková, Petr Pajer, Jiří Dresler, Ľudovít Škultéty, Marco Quevedo-Diaz

**Affiliations:** 1 Institute of Virology, Biomedical Research Center of the Slovak Academy of Sciences, Dúbravská cesta 9, 845 05 Bratislava, Slovak Republic; 2 Department of Biology, Faculty of Science, University of Hradec Kralove, Hradecká 1285, 500 03 Hradec Králové, Czech Republic; 3 Military Health Institute, Military Medical Agency, Tychonova 1, CZ-160 00 Prague 6, Czech Republic; 4 Institute of Microbiology of the Czech Academy of Sciences, Videnska 1083, 142 20 Prague 4, Czech Republic

**Keywords:** *Rickettsia akari*, Rickettsialpox, Proteome, Surface-exposed proteins, Outer membrane proteins

## Abstract

**Background:**

Rickettsialpox is a febrile illness caused by the mite-borne pathogen *Rickettsia akari*. Several cases of this disease are reported worldwide annually. Nevertheless, the relationship between the immunogenicity of *R. akari* and disease development is still poorly understood. Thus, misdiagnosis is frequent. Our study is aiming to identify immunogenic proteins that may improve disease recognition and enhance subsequent treatment. To achieve this goal, two proteomics methodologies were applied, followed by immunoblot confirmation.

**Results:**

Three hundred and sixteen unique proteins were identified in the whole-cell extract of *R. akari*. The most represented protein groups were found to be those involved in translation, post-translational modifications, energy production, and cell wall development. A significant number of proteins belonged to amino acid transport and intracellular trafficking. Also, some proteins affecting the virulence were detected. In silico analysis of membrane enriched proteins revealed 25 putative outer membrane proteins containing beta-barrel structure and 11 proteins having a secretion signal peptide sequence. Using rabbit and human sera, various immunoreactive proteins were identified from which the 44 kDa uncharacterized protein (A8GP63) has demonstrated a unique detection capability. It positively distinguished the sera of patients with Rickettsialpox from other rickettsiae positive human sera.

**Conclusion:**

Our proteomic analysis certainly contributed to the lack of knowledge of *R. akari* pathogenesis*.* The result obtained may also serve as a guideline for a more accurate diagnosis of rickettsial diseases. The identified 44 kDa uncharacterized protein can be certainly used as a unique marker of rickettsialpox or as a target molecule for the development of more effective treatment.

## Background

From the clinical and antigenic perspective, *Rickettsia* species (Order *Rickettsiales*, Family *Rickettsiaceae*) are obligate intracellular gram-negative alpha-proteobacteria that diverged into three major phylogenetic groups [1–3]. This includes the typhus group (TG), spotted fever group (SFG), and the transitional group of *Rickettsia* (TRG). *Rickettsia akari*, the causative agent of Rickettsialpox, was originally classified into the SFG. However, current genomic studies suggested placing this organism together with *R. australis* and *R. felis* into the TRG [4, 5]. This pathogen is usually transmitted to humans and animals by the rodent mites *Liponyssoides sanguineus* [6, 7]. Nevertheless, it was also detected in the mite *Leptotrombidium scutellare* [8] and Korean voles *Microtus fortis pelliceus* [9]. Rickettsialpox was first described in New York City in 1946 [10] and has been since reported in diverse parts of Europe, Asia and North America [11–16]. Patients suffering from this illness describe fever, headache, lymphadenopathy, myalgia, and eschar at the site of the mite bite. Early in the febrile course of the disease, a maculopapular eruption with intraepidermal vesicles usually appears, sparing the palms and soles of the feet [17]. But due to similar symptoms, it is often confused with cutaneous anthrax or smallpox [18, 19]. Thus, it was recommended to confirm the clinical observations with serological testing. Although a high level of cross-reactivity in antibody responses is noted between *R. akari* and other rickettsiae from the Spotted fever group [20].

Rickettsia species possess a relatively small genome (1.1 to 1.3 Mb) compared to those of their free-living relatives. Particularly, the complete genome sequence of *R. akari* comprises 1.23 megabase pairs containing 1013 protein-coding genes, 274 pseudogenes, and 39 RNA genes (gene bank accession No. CP000847). This feature is a consequence of invariable genome reduction caused by specialization to a restricted set of hosts during adaptation to the parasitic lifestyle [21]. Further analysis of rickettsial genomes, including *R. akari,* showed a number of split genes and palindromic elements inserted into genes [2]. Some data are also available from proteomic investigations of *Rickettsia* species [22–27]. The majority of identified proteins play a crucial role in the mechanism of pathogenesis and virulence of the bacteria. Proteins, however, may also act in antibiotic resistance [28] and host-specific immune response. Recent investigations on rickettsia-host interactions have also identified several important proteins involved in rickettsial adhesion and/or invasion as well as activation of host-cell signaling [29].

In this study, we investigated the antigenic potential of *R. akari* proteins using gel-free and gel-based proteomic approaches coupled to Liquid Chromatography-Mass Spectrometry (LC-MS/MS) experiments. Particular interest was paid on immunodominant cell envelope associated proteins. These key antigens might represent targets for novel diagnostics or vaccine development.

## Results

### Identification of rickettsial proteins using gel-free and gel-based proteomic approaches

Using two independent proteomics approaches, we identified 288 *R. akari* proteins in the whole bacterial lysate, from which 39 were identified as uncharacterized proteins. The identified proteins were ranging from 5.2 to 214 kDa in molecular mass and from 4.4 to 13.0 in isoelectric points. Out of these 288 proteins, 41 proteins were identified with predicted molecular masses higher than 70 kDa, 127 proteins with predicted molecular masses between 30 to 70 kDa, and 120 proteins with predicted molecular masses lower than 30 kDa. The sequence coverage of the identified proteins ranged from 1.2% (A8GLW4 – cell surface antigen) to 76.8% (A8GPB6–60 kDa chaperonin GroEL), and the abundance values expressed in label-free quantification (LFQ) ranged from 35.8 (A8GPB6–60 kDa chaperonin GroEL) to 21.5 (A8GPP7 - aspartokinase), with an average value of 26.4 (Additional file 1).

**Fig. 1:**
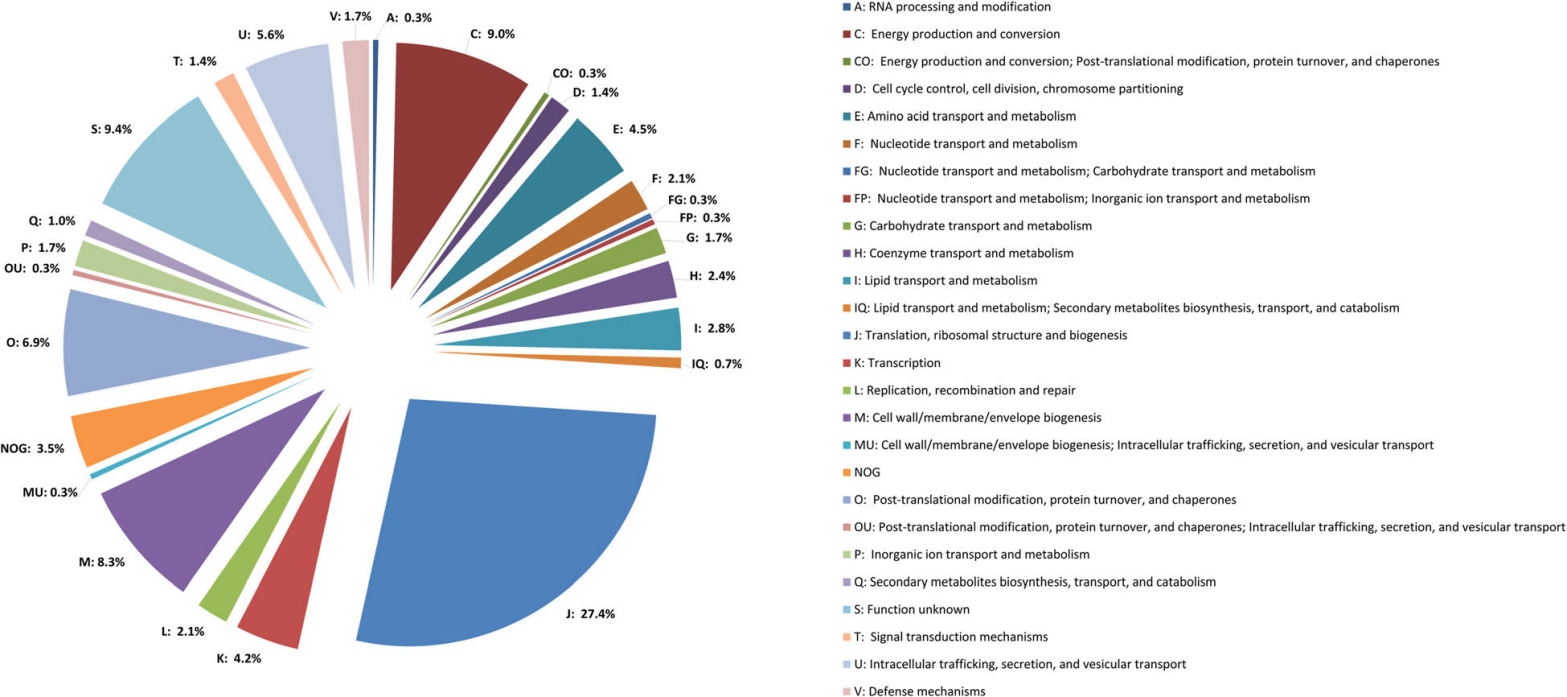
*R. akari* identified proteins assigned to their COGs. The pie chart shows the functional distribution of detected proteins by the EggNOG v5.0 database. The percentages of proteins in each COG category are indicated

The detected *R. akari* proteins were grouped into 25 distinct Clusters of Orthologous Groups (COGs), using the database EggNOG v5.0 (http://eggnog5.embl.de/#/app/home). According to this classification, 27.4% of proteins are involved in translation, ribosomal structure, and biogenesis (COG: J); 9% in energy production and conversion (COG: C); 8.3% in cell wall/membrane/envelope biogenesis (COG: M); 6.9% in posttranslational modification, protein turnover, and chaperones (COG: O); 5.6% in intracellular trafficking, secretion, and vesicular transport (COG: U); 4.5% in amino acid transport and metabolism (COG: E); 4.2% in transcription (COG: K) 9.4% with unknown function (COG: S) and 3.5% non-belong to orthologous group (NOG). The remaining 21.2% of proteins belong to 17 other COGs (A, CO, D, F, FG, FP, G, H, I, IQ, L, MU, OU, P, PQ, T, V) in the share of 0.3 to 2.8% (Fig. 1).

### Surface-exposed and envelope associated proteins (SEPs)

SEPs of *R. akari* represent an essential interface in pathogen-host interactions. To recognize these proteins, two membrane-enriched rickettsial extracts were prepared as detailed in the materials and method section. In the first, the cell-impermeant reagent, Sulfo-NHS-LC-Biotin, was used to label SEPs. The biotinylated proteins were then isolated by streptavidin agarose affinity purification. On the other hand, the second protocol was based on the Triton X-114 phase partitioning. This non-ionic detergent substitutes the lipid molecules interacting with the hydrophobic domain of integral membrane proteins and forms soluble protein–detergent micelles. After temperature changes, phase separation occurs. Hydrophobic proteins aggregate in the detergent phase, while the hydrophilic remain in the aqueous layer [30]. All the obtained fractions were then separated by 12% SDS-PAGE and analyzed with LC-MS/MS. Using these approaches¸ we identified 83 unique *R. akari* proteins with molecular masses range from 5.5 to 168.0 kDa and p*I* from 4.6 to 12.1. Fourteen of these proteins were recognized with both approaches, including OmpB (A8GPL7), chaperone protein DnaK (A8GMF9), 60 kDa chaperonin GroEL (A8GPB6), ATP synthase subunit alpha (A8GPZ6), a 44 kDa uncharacterized protein (A8GP63), superoxide dismutase (A8GNP0), putative adhesin A1C_06425 (A8GQ33), thioredoxin peroxidase 1 (A8GN15), inorganic pyrophosphatase (A8GP57), stress-induced DNA-binding protein (A8GPZ9), nucleoside diphosphate kinase (A8GLZ8), 50S ribosomal protein L7/L12 (A8GMA6), 10 kDa chaperonin (A8GPB7) and 7 kDa uncharacterized protein (A8GNR0).

**Fig. 2:**
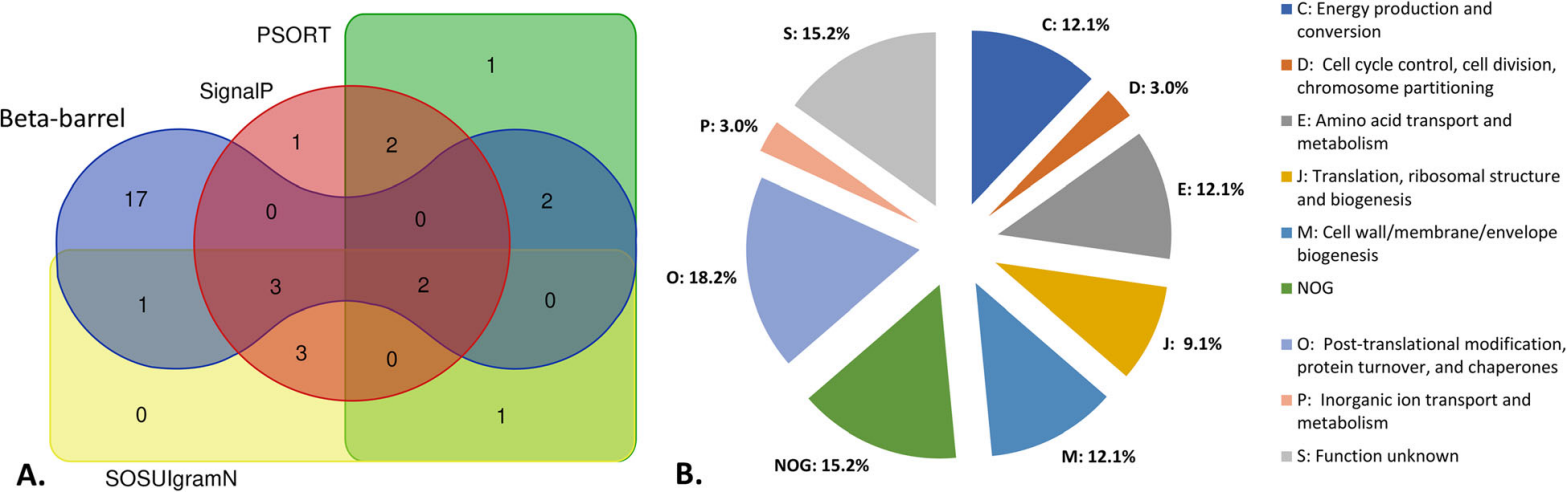
*R. akari* SEPs detected in the membrane enriched fractions. (**a**) Venn diagram of proposed SEPs using four different prediction tools. (**b**) Pie chart illustrating the distribution of R. akari SEPs identified in membrane protein-enriched fractions among the COGs

Out of the 83 identified proteins, 15 were predicted using the PSORTb prediction tool [31] as a membrane or secreted extracellular proteins (Fig. 2a, Table 1), 1 protein as periplasmic, 48 proteins as cytoplasmic, and 17 proteins with unknown followed by 2 with multiple localizations. Using the SOSUIgramN program [32], 20 proteins were anticipated as a membrane or extracellular (Fig. 2a, Table 1), 2 proteins as periplasmic, 57 proteins as cytoplasmic and 4 proteins with unknown locations. In addition, the PRED-TMBB online tool [33] recognized 25 proteins with beta-barrel structure (Fig. 2a, Table 1), and 11 proteins were predicted with the Signal P–5.0 server to possess a signal peptide at the N-terminus (Fig. 2a, Table 1). Interestingly, nine of these proteins apparently carry a Type I secretory signal peptides that are usually transported by the Sec or twin-arginine translocon (Tat). These proteins include the OmpB (A8GPL7), 44 kDa uncharacterized protein (A8GP63), putative adhesin A1C_06425 (A8GQ33), putative surface antigen (A8GM15), 19 kDa uncharacterized protein (A8GP34), 17 kDa uncharacterized protein (A8GPM2), tail-specific protease (A8GMM4), protein export protein PrsA (A8GP43), and the 18 kDa uncharacterized protein (A8GNE2). Additional two proteins, namely 9 kDa uncharacterized protein (A8GNC4) and a peptidoglycan-associated lipoprotein (A8GPW0), were predicted to have a lipoprotein signal peptides suggesting Type II secretion.

**Table 1:** Surface exposed proteins of *R. akari*

Uniprot Accession Number	Description	mW (KDa)	pI	Identified by:	Subcellular localization		Predicted Motifs		Immunogenic
					PSORTb	SOSUIgramN	β-barrel score^a^	Signal P Type (Likelihood)	
A8GQ33	Putative adhesin A1C_06425	24.1	9.7	B/T/S	outer membrane	outer membrane	2.819^a^	Sec/SPI (0.9982)	–
A8GMA6	50S ribosomal protein L7/L12	13.0	5.0	B/T/S	multiple	cytoplasmic	2.851^a^	no	–
A8GPL7	Outer membrane protein B	167.9	5.0	B/T/S	outer membrane	extracellular	2.861^a^	Sec/SPI (0.3494) Tat/SPI (0.5171)	yes
A8GPB6	60 kDa chaperonin	58.7	5.3	B/T/S	cytoplasmic	cytoplasmic	2.886^a^	no	yes
A8GM15	Putative surface antigen	48.1	9.6	B	inner membrane	extracellular	2.898^a^	Sec/SPI (0.9979)	–
A8GN82	Malate dehydrogenase	33.6	5.4	T /S	cytoplasmic	cytoplasmic	2.901^a^	no	–
A8GN16	Membrane protease subunits	33.9	5.5	T /S	unknown	inner membrane	2.902^a^	no	–
A8GPV8	Rod shape-determining protein MreB	37.4	5.3	T /S	cytoplasmic	cytoplasmic	2.912^a^	no	–
A8GMZ9	ATP-dependent protease subunit HslV	19.8	6.7	T	cytoplasmic	cytoplasmic	2.932^a^	no	–
A8GM33	Elongation factor Ts	33.7	5.2	T /S	cytoplasmic	cytoplasmic	2.932^a^	no	–
A8GM25	Preprotein translocase subunit SecG	6.5	9.5	B	unknown	unknown	2.936^a^	no	–
A8GMB0	Probable cytosol aminopeptidase	53.3	6.0	T /S	cytoplasmic	cytoplasmic	2.939^a^	no	–
A8GPZ4	ATP synthase subunit beta	51.0	4.6	T /S	multiple	cytoplasmic	2.940^a^	no	–
A8GP69	Phospho-N-acetylmuramoyl-pentapeptide-transferase	39.7	8.4	B	inner membrane	inner membrane	2.946^a^	no	–
A8GPZ6	ATP synthase subunit alpha	56.2	6.4	B/T/S	cytoplasmic	cytoplasmic	2.947^a^	no	–
A8GNC4	Uncharacterized protein	9.1	8.6	B/S	unknown	outer membrane	2.948^a^	Sec/SPII (0.9985)	–
A8GMH0	2,3,4,5-tetrahydropyridine-2,6-dicarboxylate N-succinyltransferase	30.0	7.0	T /S	cytoplasmic	cytoplasmic	2.950^a^	no	–
A8GMS9	Heat shock protein	18.6	9.2	T /S	unknown	outer membrane	2.954^a^	no	–
A8GP63	Uncharacterized protein	44.6	8.5	B/T/S	unknown	extracellular	2.957^a^	Sec/SPI (0.9890)	yes
A8GMF9	Chaperone protein DnaK	67.7	4.8	B/T/S	cytoplasmic	cytoplasmic	2.960^aa^	no	yes
A8GNU1	Aminotran_5 domain-containing protein	40.7	6.7	T	cytoplasmic	cytoplasmic	2.961^a^	no	–
A8GLV8	ATP synthase subunit a	27.3	9.0	B	inner membrane	inner membrane	2.962^a^	no	–
A8GNF1	4-hydroxy-tetrahydrodipicolinate synthase	32.4	7.2	T	cytoplasmic	cytoplasmic	2.962^a^	no	–
A8GPZ9	Strees induced DNA-binding protein	16.1	4.9	B/T/S	cytoplasmic	cytoplasmic	2.963^a^	no	–
A8GLX8	50S ribosomal protein L9	19.4	7.7	B/S	cytoplasmic	cytoplasmic	2.964^a^	no	–
A8GML3	Uncharacterized protein	61.3	5.5	T /S	outer membrane	inner membrane	2.986	no	–
A8GP43	Protein export protein prsA	31.4	9.4	T /S	outer membrane	cytoplasmic	2.989	Sec/SPI (0.9958)	–
A8GP34	Uncharacterized protein	19.5	5.7	B/S	unknown	extracellular	2.990	Sec/SPI (0.9796)	–
A8GMM4	Tail-specific protease	50.1	7.3	T /S	inner membrane	outer membrane	2.990	Sec/SPI (0.8945)	–
A8GNE2	Uncharacterized protein	18.8	9.0	T	unknown	periplasmic	2.993	Sec/SPI (0.9866)	–
A8GPM2	Uncharacterized protein	17.0	6.2	B/S	unknown	extracellular	3.006	Sec/SPI (0.9979)	–
A8GP67	Actin polymerization protein RickA	59.6	10.1	T /S	outer membrane	extracellular	3.035	no	–
A8GPW0	Peptidoglycan-associated lipoprotein	17.5	8.9	T /S	outer membrane	unknown	3.106	Sec/SPII (0.9996)	yes

SEPs detected using, *B* biotinylation, *T* Triton X-114, and/or *S* Shotgun proteomic approaches. ^a^values below the threshold 2.965 indicate the presence of beta-barrel structure

Using the four bioinformatics algorithms, we suggested 33 proteins with predicted outer membrane localization or possessing signal peptides or beta-barrel structure as *R. akari* SEPs (Table 1). These proteins were then grouped into 8 distinct COGs, using the database EggNOG v5.0. According to this classification, 18.2% of proteins are involved in posttranslational modification, protein turnover, and chaperones (COG: O); 15.2% with unknown function (COG: S); 12.1% in cell wall/membrane/envelope biogenesis (COG: M); 12.1% in energy production and conversion (COG: C); 12.1% in amino acid transport and metabolism (COG: E); 9.1% of proteins are involved in translation, ribosomal structure, and biogenesis (COG: J). The remaining 6% of proteins belong to 2 other COGs (D and P) in the share of 3% each (Fig. 2b, Additional file 2). Five proteins were not classified into any clusters of orthologous groups due to unknown functions.

### Immunogenic SEPs

**Fig. 3:**
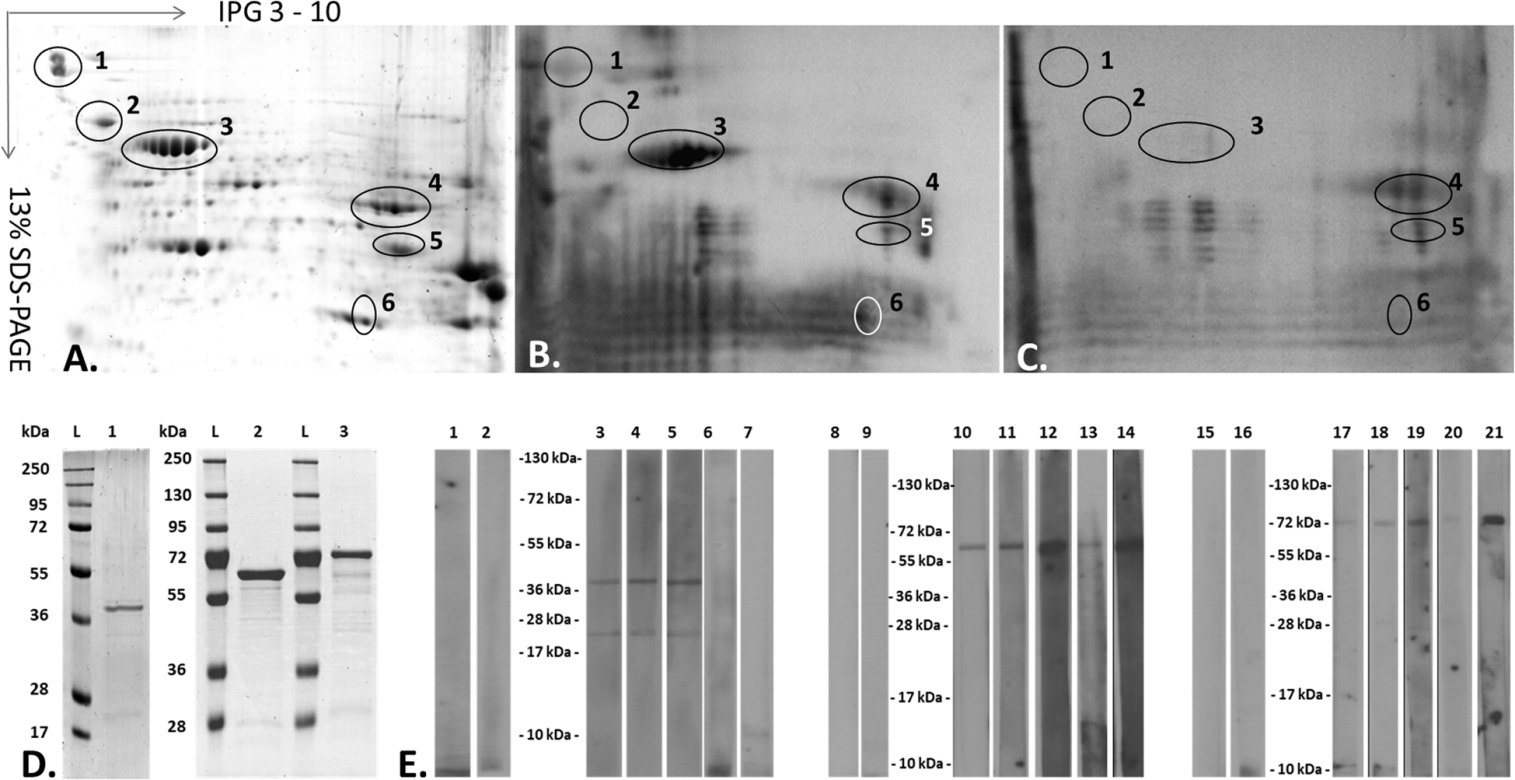
Immunogenic SEPs of *R. akari*. (**a**) *R. akari* proteins (140 μg) from whole-cell extract separated by 2-DE. 1- OmpB (A8GPL7), 2 - chaperon protein DnaK (A8GMF9), 3–60 kDa chaperonin GroEL (A8GPB6), 4–44 kDa uncharacterized protein (A8GP63), 5 – peptidoglycan associated lipoprotein (A8GPW0), 6 – superoxide dismutase (A8GNP0) (**b**) 2-D Western blot analysis of *R. akari* proteins using serum from infected rabbit (1:1000) (**c**) or infected patient’s serum (1:1000) (**d**) SDS-PAGE of selected SEPs prepared with recombinant technology. L: protein marker; lane 1–44 kDa uncharacterized protein (A8GP63) (3 μg); 2–60 kDa chaperonin GroEL (8 μg); 3- chaperon protein DnaK (6 μg). (**e**) Western blot analyses of recombinant (2 μg per lane) 44 kDa uncharacterized protein (lanes 1–7), 60 kDa chaperonin GroEL (lanes 8–14), and chaperon protein DnaK (lanes 15–21) probed against - 1-2, 8–9, 15–16: sera of healthy donors (negative controls, 1:1000); 3–5, 10–12, 17–19: sera of Rickettsialpox patients (1:1000), and 6–7, 13–14, 20–21: sera of patients with SFG rickettsial infection (1:1000)

In order to identify antigenic proteins, the 2-dimensional electrophoresis (2-DE, Fig. 3a) of the whole *R. akari* cell extract were probed against the anti - *R. akari* polyclonal rabbit serum (Fig. 3b) and the serum of infected patient clinically diagnosed with rickettsialpox (Fig. 3c). From the identified immunoreactive proteins, five were recognized in this study as SEPs, namely a 60 kDa chaperonin GroEL (A8GPB6), OmpB (A8GPL7), DnaK (A8GMF9), Peptidoglycan-associated lipoprotein (A8GPW0), and a 44 kDa uncharacterized (A8GP63) protein.

The 44 kDa uncharacterized protein (A8GP63) together with the highly abundant 60 kDa (GroEL) (A8GPB6) and the DnaK (A8GMF9) were prepared by ectopic expression in *E.coli* BL21(DE3). Purified recombinant proteins were then separated by SDS-PAGE (Fig. 3d) and probed against five sera from patients with confirmed rickettsial infection (2 sera of patients with SFG rickettsiosis and 3 with Rickettsialpox) (Fig. 3e). Interestingly, the proteins GroEL and DnaK reacted with all the patient’s sera. However, the 44 kDa uncharacterized protein (A8GP63) conducted a response only with the sera of patients suffering from Rickettsialpox (Fig. 3e, lanes 3, 4, 5). No reactivity was observed with the sera of patients with SFG rickettsiosis. In this protein preparation, two bands (about 44 kDa and 22 kDa) were seen in the SDS-PAGE and the immunoblots. However, both correspond to the 44 kDa uncharacterized protein as determined by LC-MS /MS analysis. The lower and upper ones represent the truncated and full-length protein, respectively (Fig. 3c).

### Characterization of the 44 kDa uncharacterized protein (A8GP63)

**Fig. 4:**
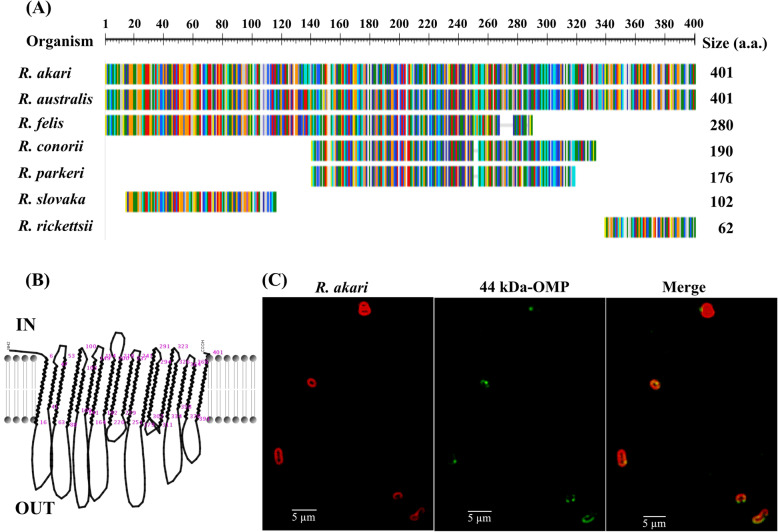
The 44 kDa protein (A8GP63) of *R. akari* as an outer membrane protein. (**a**) Homology search to 44 kDa protein using blast analysis (**b**) In silico prediction of the protein secondary structure (**c**) IFA using a “pre-absorbed” mouse polyclonal antibody against the 44 kDa protein

The homology searches for the *R. akari* putative 44 kDa uncharacterized protein against the NCBI database showed high identity (91.8%) to uncharacterized protein from *R. australis* and 83.2% to unknown protein (RF_0375) from *R. felis* (accession number AAY61226.1). However, only a low level of sequence similarity and protein size was noted in the comparison to other SFG rickettsiae (e.g., *R. conorii* (AAL03440.1), *R. parkeri* (WP_146709264.1), *R. slovaca* (WP_041472097.1), *R. rickettsii* (WP_041472549.1) (Fig. 4a). Such a drift usually occurs due to gene loss which is a major source of genome diversification within *Rickettsia* species and may reflect host specialization to distinct arthropods [16].

Based on precise bioinformatics predictions, we could suggest that this 44 kDa protein is having a Sec signal peptide (Sec/SPI) and secondary transmembrane structure (Fig. 4b) that is represented by beta-barrels typical for outer membrane proteins. The protein structure also showed a high percentage of total accessible surface area that could be easily available for antibody binding. Using the B-cell type epitope predictor, we revealed the candidate epitopes for the 44 kDa uncharacterized protein (A8GP63). We found 10 potential peptides that showed a score higher than the cut-off. Six of them were exposed on the protein surface and probably well available for antibody binding (Additional file 3). Thus, this observation may suggest that the *R. akari* protein can be involved in the specific interaction with the host cell.

To confirm surface localization of this protein we performed a dual immunofluorescence antibody assay. For this purpose, we developed a mouse polyclonal antibody against the 44 kDa uncharacterized protein and conjugated it with Alexa 488 which provides a green fluorescence signal. In order to significantly reduce cross-reactivity and to increase the specificity of protein recognition, we pre-absorbed the mouse serum with *E. coli* cells prior to immunofluorescence assay (IFA). As seen in Fig. 4c, the green fluorescence signal is apparently associated with the outer membrane of *R. akari*. We observed a robust protein accumulation clustering near the pole of rickettsial cells. Such a feature was also recorded for surface cell antigens (Sca) of *R. typhi*, including Sca2, Sca3, and Sca4, as seen in immunoelectron microscopy where clustering of Sca proteins on the surface of intact rickettsia was clearly demonstrated [34].

## Discussion

In this work, a comprehensive proteomic study of *R. akari* was performed using gel-based and gel-free approaches with the aim to identify proteins having a key role in pathogen-host interactions. In the first step, proteins from the whole-cell extract of the bacterium were separated by 2-DE. In the gel (Fig. 3a), 356 protein spots were detected. The spots were excised and identified by ESI-LC-MS/MS mass spectrometry. Obtained data were then combined with protein identification from a gel-free (shotgun) proteomic approach that was based on LC-MS/MS analyses. These proteomic analyses resulted in the identification of 288 unique proteins that correspond to 28.4% of 1013 protein-coding genes annotated in the *R. akari* genome. Similar numbers of proteins were identified in previous proteomic studies in which *R prowazekii, R. conori, R. raoultii, R. massiliae, R. slovaca,* and *R. parkeri* were evaluated [22, 24–26, 35].

The largest group of identified proteins was associated with translation, ribosomal structure, and biogenesis. This group was tightly followed by enzymes involved in the processes of central metabolic pathways such as glycolysis, pentose phosphate pathway, tricarboxylic acid (TCA) cycle, oxidative phosphorylation, fatty acid metabolism, and amino acid metabolism. Four key enzymes of TCA cycle, which have been shown to be complete in *Rickettsiae* (KEGG, MAP 00020) were detected in this study, namely malate dehydrogenase (A8GN82) citrate synthase (A8GQ56), acetyltransferase component of pyruvate dehydrogenase complex (A8GNQ3), and 2 subunits (alpha and beta) of pyruvate dehydrogenase E1 component (A8GMR3 and A8GMR4 respectively). Despite the evidence of a functional presence of pyruvate dehydrogenase complex and TCA cycle, it was shown that *Rickettsia spp*. cannot metabolize (glycolysis) or generate (gluconeogenesis) glucose [36]. Neither pyruvate can be synthesized due to the lack of metabolic pathways necessary for pyruvate synthesis [37]. Therefore, glucose and pyruvate should be uptaken directly or in the form of phosphoenolpyruvate from the host cells cytoplasm to support ATP generation via the TCA cycle [23]. In this case, phosphoenolpyruvate is converted to pyruvate by pyruvate phosphate dikinase (A8GNN4), which was detected in this study. Nevertheless, ATP can be synthesized by oxidative phosphorylation. The presence of four ATP synthase subunits (A8GPZ3, A8GPZ4, A8GPZ6, A8GPZ7) illustrates the importance of this pathway in *Rickettsiae* that is necessary to compensate for glycolysis defect [23].

In addition to exogenous uptake of major energy sources including glucose, ATP and NAD^+^ [36], *Rickettsiae* as intracellular organisms possess refined secretion mechanisms to translocate various effector proteins into the host cell in order to sustain survival. They are equipped with various types of secretion systems, including the Sec or Tat translocation pathways, type I secretion system (T1SS), type IV secretion system (T4SS), and type V autotransporter pathways (T5SS) [38]. As in earlier rickettsial proteome studies [22, 25], we identified several proteins of Sec-mediated translocation system, namely protein translocase subunit SecD (A8GP54), Sec translocon accessory complex subunit YajC (A8GP53), signal recognition particle receptor FtsY (A8GPW4), preprotein translocase subunit SecG (A8GM25), and a protein-export membrane protein SecG (A8GM27), that is unique to all rickettsiae [23, 25]. We also identified two out of the three major structural features of the T1SS, namely outer membrane efflux protein (A8GMM0) belonging to the TolC family [38] and a putative ABC transporter ATP-binding component (A8GM03). This protein is a constituent of ATP-binding cassette transporters that play a role in multidrug resistance [23, 39]. Moreover, we detected three components of T4SS, namely TrbL/VirB6 plasmid conjugative transfer proteins (A8GM63–67), VirB4 family protein (A8GM62), and VirD4 protein (A8GMV1). The last interact with a VirB4 protein and the ATPases VirB11 independently of other subunits and forms the ATPase complex that dock to the inner membrane. The VirD4 also acts as a receptor that may transport substrates to the translocation channel [40, 41]. Meanwhile, VirB6 is the key polytopic membrane protein forming the inner membrane channel that is essential for substrate secretion (Additional file 1).

Secreted proteins and SEPs play a key role in pathogen-host interactions by allowing infection of the host cell and promoting bacterial replication in the cytosol [34]; hence identification of these important molecules is essential. Obviously, the surface cell antigen (Sca) family proteins are undoubtedly included among the most dominant SEPs which play an important role in rickettsial pathogenesis [42–44]. Previous studies indicated that two major Sca proteins, outer membrane protein A (Sca0 or OmpA) and OmpB (Sca5), might play key roles in the adhesion of the *Rickettsia* cells to the host tissue [42, 44–46]. These molecules are likely major antigenic determinants of *Rickettsia spp* and represent good candidates for vaccine development. Nucleotide sequences of these proteins were even used for phylogenetic determination. While OmpB (A8GPL7) has been identified in several *Rickettsia* spp. including *R. akari* (in this study), the presence of OmpA was reported only in the SFG [47]. The latest protein is mostly not encoded in the genomes of TG *Rickettsia* [34]. Further evaluation of the identified proteins has revealed many similar SEPs as in other *Rickettsiae,* including the OmpB (A8GPL7), putative adhesin (A8GQ33), heat shock protein (A8GMS9), and chaperone proteins DnaK (A8GMF9) and GroEL (A8GPB6). Using enrichment protocols, we also detected the cell division coordinator CpoB (A8GMM6) surface protein, the TolB protein (A8GMX9), and the actin polymerization protein RickA (A8GP67). The CpoB was previously described as a YbgF protective antigen [27]. Together with TolB, these periplasmic proteins are essential for the Tol-Pal system, which has a role in the maintenance of cell envelope integrity. These proteins may also participate in translocation of virulence factors [48]. On the other hand, the identification of actin polymerization protein RickA (A8GP67), an activator of the host Arp2/3 complex, may indicate the actin-based motility of *R. akari* [49, 50]. Thus this protein could be proposed as an important factor of virulence in SFG *Rickettsia*. It promotes spreading among host cells, unlike the Typhus group members [51, 52].

However, the main aim of this study was to investigate the antigenic potential of these important *R. akari* proteins. For this propose, the whole-cell extract was analyzed using a two-dimensional immunoblot technique coupled to LC-MS/MS identification. Under our experimental conditions, a strong immunological response of *R. akari* lipopolysaccharide against the polyclonal sera of infected rabbit and positive patient sera was evident. Nevertheless, among the proteins with predicted beta-barrel structure, which were proposed to be *R. akari* SEPs, we detected only four immunoreactive antigens, namely chaperone proteins GroEL (A8GPB6) and DnaK (A8GMF9), outer membrane protein B (A8GPL7), and an uncharacterized protein (A8GP63). Heat shock proteins GroEL and DnaK are the most frequently detected immunoreactive proteins with high abundance in *Ricketssiae,* as it was indicated in many previous studies on *R. felis*, *R. conorii, R. helvetica*, and *R. rickettsii* [24, 25, 53]. The upregulation of *groEL* and *dnak* was also described in *R. prowazekii* as a response to heat shock exposure [54]. According to our shotgun label-free proteomic quantitation (LFQ), proteins GroEL and DnaK belong to the most abundant proteins of *R. akari*, with LFQ values 35.8 and 32.2, respectively, whereas the average value of all identified proteins achieved 26.4. Interestingly, one of the uncharacterized proteins, the 44 kDa uncharacterized protein (A8GP63) also seems to be abundant as it reached the LFQ value of 30.1.

So far, no evidence was published concerning this uncharacterized protein (A8GP63). The protein homology search showed very high sequence similarity with two unknown orthologous proteins from *R. australis* (H8K6W8) and *R. felis* (Q4UMI2). However, only a very low similarity was noted to other SFG rickettsiae (Fig. 4a). This is in line with the classification to TRG of *Rickettsia* since *R. akari* is phylogenetically related the most closely to *R. australis* and *R. felis* [4]. To better describe this unique protein, it was prepared by ectopic expression in *E. coli*. At the same time, we also produced the highly abundant 60 kDa (GroEL) and 70 kDa (DnaK) of *R. akari* for comparative purposes. Then, these recombinant proteins were tested against 3 positive sera from Rickettsialpox patients and 2 positive patient sera with spotted fever group rickettsiosis. As we predicted, all positive patient sera distinguished the recombinant proteins GroEL and DnaK. However, the 44 kDa unknown protein was recognized exclusively with the positive sera from Rickettsialpox patients. Moreover, IFA using specific mouse serum against this 44 kDa uncharacterized protein (A8GP63) together with precise bioinformatics suggested membrane association. Thus, the 44 kDa uncharacterized protein (A8GP63) appears to be a valuable biomarker of *R. akari.*

## Conclusions

This paper represents the first comprehensive proteomic study of *R. akari* that was accomplished using gel-based and gel-free proteomics approaches. Altogether we identified 316 unique proteins that correspond to 31.2% of 1013 protein-coding genes annotated in the *R. akari* genome. From them, 33 proteins found in the cell envelope enrichment fractions were predicted as outer membrane proteins or proteins possessing a signal peptide or a beta-barrel structure indicating membrane association. These proteins were proposed as *R. akari* SEPs that may play a key role in pathogen-host interactions. However, antigenic protein seems to be the most important in terms of effective vaccine development and the discovery of new biomarkers for clinical diagnosis. Among these proteins, we recognized an immunodominant 44 kDa uncharacterized protein (A8GP63) that was not yet described in any *Rickettsiae*. It seems this protein has beta-barrel structures indicating outer membrane character which was proved by IFA. In addition, the protein shows significant immunoreactivity against the sera of patients with Rickettsialpox. Thus, the 44 kDa putative outer membrane protein (A8GP63) represents a good candidate for improved differential diagnosis of rickettsial diseases especially those caused by TRG of *Rickettsia*.

## Methods

### Bacterial growth and purification of *R. akari*

*R. akari* reference strain Hartford was propagated in egg yolk sacs of pathogen-free chicken embryos in BSL-3 containment as described previously [55] with some modifications. Briefly, 6 days old chicken embryos were inoculated and incubated at 35 °C. The bacterial inoculum was adjusted; therefore, most embryos died between 8 to 9 days after inoculation. Yolk sacs were collected, homogenized in 2 M NaCl solution and the suspension was centrifuged at 22000 x g at 4 °C. Pellet was resuspended in PBS and centrifuged again at low speed (200 x g for 10 min) to remove debris. The supernatant was then overlaid on 25% w/w sucrose cushion and centrifuged at 22000 x g at 4 °C. Resulted pellet was re-suspended in sucrose phosphate glutamate (SPG) buffer and purified by two rounds of Ultravist 370 (Bayer, Germany) discontinuous gradient centrifugation by using 32, 36, and 42% layers at 90000×g at 4 °C for 50 min. Light bands containing intact bacteria were collected, and centrifuged at 22000 x g for 30 min [55]. Finally, the pellet was re-suspended in PBS and stored at − 80 °C until analysis. Giménez staining technique was employed to evaluate the purity of rickettsial cells.

### Gel-free proteomics

The bacterial cells of *R. akari* (4 mg/mL) were pelleted by centrifugation (18,000 x g; 20 min; 4 °C) and washed with 300 μl PBS. The resulting pellets were re-suspended in 100 μl of 50 mM Tris pH 7.5 containing 0.1% RapiGestTM SF (Waters, UK) [56] and then incubated for 10 min at 95 °C. After cooling, 200 μl of 0.1% RapiGestTM SF in 8 M guanidinium chloride (Sigma-Aldrich, USA) was added and incubated for another 20 min. Then, filter aided sample preparation – (FASP) [57] protocol was applied. Briefly, inactivated samples were transferred onto Amicon® Ultra – 10 kDa filters (Millipore) and washed twice with 100 mM ammonium bicarbonate (Sigma-Aldrich). Subsequently, proteins were quantified by bicinchoninic acid assay (QuantiPro™ BCA Assay Kit, Sigma-Aldrich) [58]. The samples were then reduced with 100 mM Tris (2-carboxyethyl) phosphine hydrochloride (TCEP, Sigma-Aldrich) and alkylated with 300 mM iodoacetamide (Sigma-Aldrich). Finally, the samples were digested with 2 μg of sequencing grade trypsin (Promega) overnight at 37 °C. Empore™ SPE Cartridges, C18, standard density, bed I.D. 4 mm (Sigma-Aldrich) were used to desalt peptide mixtures before drying to completion in a speed-vac. Prior to mass spectrometry analysis, the samples were re-suspended in 30 μl of 2% acetonitrile (ACN)/0.1% trifluoroacetic acid.

The samples were further analyzed by Ultimate 3000 RSLCnano system controlled by Chromeleon software (Dionex, USA), involving targeted mass spectrometry and LFQ as described earlier [59] with some modifications. Briefly, extracted peptide mixtures were loaded onto a PepMap100 C18, 3 μm, 100 Å, 0.075 × 20 mm trap column (Dionex) at 5 μl/min for 5 min. Separation was performed on a PepMap RSLC C18 column (0.075 × 150 mm, particle size 2.0 μm; Dionex) using 68 min gradient of 4–34% mobile phase B (80% ACN, 0.1% FA) with mobile phase A (0.1% formic acid, FA) and 21 min gradient of 34–55% mobile phase B at flow rate of 0.3 μl/min. Eluted peptides were electrosprayed into a Q-Exactive mass spectrometer using a Nanospray Flex ion source (Thermo Scientific, Bremen, Germany) to obtain positive ion full-scan MS spectra in the range 350–1650 m/z.

Acquired raw files were further processed in MaxQuant (version 1.6.7.0) [60]. Andromeda search engine [61] software was applied to identify proteins against the *Rickettsia akari* strain Hartford databases downloaded from Uniprot (September 21st, 2019). Identifications were accepted if at least two distinct reliable peptides matched the protein sequence, or the sequence coverage achieved at least 15%. Relative quantification was performed using the default parameters of the MaxLFQ algorithm [62], with the minimum ratio count set to 2.

### Protein preparation and 2-D electrophoresis (2-DE)

The rickettsial pellet (2 mg wet-weight) was re-suspended in 4 mL of lysis buffer [28 mM Tris-HCl, 22 mM Tris-base, 200 mM dithiothreitol (DTT, Promega, USA), 2% SDS, protease inhibitor] and incubated for 30 min at 4 °C under shaking. After incubation sample was boiled for 5 min at 100 °C and cooled down on the ice for 5 min, followed by incubation with benzonase nuclease (Sigma-Aldrich, USA) 1ul/mL during 1 h at room temperature. The supernatant was collected after centrifugation at 14000 x g for 20 min at 4 °C and proteins precipitated with an equal volume of chloroform, 5 volumes of methanol, and 3 volumes of MilliQ water was added prior centrifugation at 14000 x g for 5 min at 4 °C. After phase separation, the upper phase was discarded, the same volume of methanol was added and vortexed. The protein pellet was collected by centrifugation at 14000 x g for 20 min at 4 °C, dried with N_2_ and stored at − 80 °C. Samples were dissolved in lysis buffer (8 M urea, 2 M thiourea, 85 mM DTT, 2.5% Triton X-100) prior to rehydration.

The IPG strip (18 cm, pH 3–10 NL; GE Healthcare, USA) was rehydrated overnight with approx. 140 μg of total protein with 1.0% v/v IPG Buffer (GE Healthcare) and 0.001% bromophenol blue in buffer No.7. The isoelectric focusing (IEF) was carried out in the Ettan IPGphor 3 IEF System apparatus (GE Healthcare, USA). After IEF each IPG strip was incubated in equilibration buffer composed of 6 M urea, 2% SDS, 30% glycerol, 375 mM Tris-HCl (ph 8.8) in the presence of 1.0% DTT for 15 min, followed by equilibration for 15 min in 3.75% iodoacetamide (IAA, Sigma-Aldrich, USA) solution in the same equilibration buffer. Equilibrated strips were transferred onto 13% polyacrylamide gels to perform second dimension SDS-PAGE. After electrophoresis, gels were stained with Coomassie brilliant blue R-250 (Serva, Germany) and stored at 4 °C until spot excision and trypsin digestion.

### In-gel peptide digestion and MS analysis

Protein spots were excised, transferred into 1.5 mL Eppendorf tubes and distained in 50 mM ammonium bicarbonate buffer containing 50% acetonitrile (ACN, Merck, Germany), reduced with 10 mM DTT in 100 mM ammonium bicarbonate, followed with alkylation in 50 mM IAA in 100 mM ammonium bicarbonate buffer [63]. Proteins were digested with porcine trypsin in 10 mM ammonium bicarbonate with 10% acetonitrile at 37 °C overnight. The enzymatic reaction was stopped with a 70% acetonitrile solution containing 1.0% trifluoroacetic acid (TFA, Merck, Germany), and peptides were subsequently extracted. Supernatants were concentrated on SpeedVac (Eppendorf, Germany) to a final volume of 20 μl and analyzed by automated nanoflow reverse-phase (RP) liquid chromatography coupled to a Q-TOF (quadrupole time-of-flight) Premier Electrospray Ionisation (ESI) tandem mass spectrometer (Waters, USA). Separation of peptides was carried out on RP column BEH 130 C18 (200 mm × 75 μm, particle size 1.7 μm; Waters) using a 60 min gradient elution of 5–40% acetonitrile with 0.1% (w/w) formic acid at a flow rate of 0.3 μl/min. Samples were nanosprayed at 3.4 kV capillary voltage to Q-TOF detector and spectra were recorded from alternate scans at low (4 eV) and high (20–40 eV ramp) collision energies to obtain full-scan mass in ion range 50–1950 m/z. Finally, the data were processed by ProteinLynx Global Server 3.0.3 (Waters) searching the UniProt database (https://www.uniprot.org/proteomes/UP000291740). For peak picking the following thresholds were applied: low energy 140 counts and high energy 30 counts. Precursors and fragment ions were coupled, using correlations of chromatographic elution profiles in low/high energy traces. Spectra were searched against the assembled *Rickettsiaceae* proteome database (downloaded on January 20th, 2019). Workflow parameters for the protein identification queries were: i) maximum one possible trypsin miscleavage; ii) a fixed carbamidomethyl cysteine, variable oxidized methionine and deamidated asparagine/glutamine; iii) the precursors and fragments mass tolerance was automatically determined by the software; iv) peptide matching was limited to less than 4% false discovery rate against the randomized database. Identifications were accepted if at least two distinct reliable peptides matched the protein sequence or the sequence coverage achieved at least 15%.

### Membrane protein enrichment methods

The first method was based on the Triton X-114 phase separation [30]. Briefly, the purified *R. akari* cells were resuspended in Triton X-114 extraction solution (1% Triton X-114, 10 mM Tris pH 7.5, 5 mM EDTA) and incubated on rotating platform at 4 °C for 4 h. After the extraction¸ sample was centrifuged at 15000 x g at 4 °C for 15 min, resulting in cell debris containing pellet and supernatant. This supernatant was layered over a chilled sucrose cushion, incubated at 37 °C for 30 min, and centrifuged at 500 x g for 20 min at 30 °C to separate the lower detergent and the upper aqueous phase. The proteins from the Triton X-114 and aqueous fractions were precipitated in acetone at − 20 °C prior to separation.

As a second membrane enrichment technique, the cell surface biotinylation method was applied. Briefly, purified *R. akari* bacteria were labeled with Sulfo-NHS-LC-Biotin (Thermo Scientific, USA) and the biotinylated proteins were captured on streptavidin agarose resin (Thermo Scientific) as described previously [64]. The captured proteins were eluted from the streptavidin resin with a 5% solution of 2-mercaptoethanol-PBS at 30 °C for 30 min, repeated 3 times, and the eluted proteins were precipitated in acetone at − 20 °C overnight. All protein fractions were then dissolved in Laemmli sample buffer and subjected to SDS-PAGE on 12% polyacrylamide gel. The whole lane was cut and processed by in-gel trypsin digestion and analyzed by LC-MS/MS as described above.

### Preparation of recombinant proteins

The genes *groEL*, *dnaK* and 44 kDa uncharacterized protein (locus: A1C_04610) were amplified from the genomic sequence of *R. akari* (GenBank accession number: CP000847) with *groEL* primer pairs, 5′- TATA-CCATGG (*Nco*I)-CAACAAAACTTATTAAACACG − 3′ and 5′- TATA-AGATCT (*Bgl*II)-GGAAATCCATACCGCCCATA − 3′ and *dnaK* primer pairs, 5′- TATA-CCATGG (*Nco*I)-GAAAAGTAATAGGTATTGACCTTGG − 3′ and 5′- TATA-AGATCT (*Bgl*II)-TCTTCTTCGCTACATCCTGAAAATCG − 3′ and 44 kDa uncharacterized protein (locus: A1C_04610) primer pairs, 5′- AG-CCATGG (*Nco*I)-GTAAATTAAATAAATTAAATTTAACTATTGC − 3′ and 5′- CTGCAG (*Pst*I)-CTAAATCTAATTTTAACCCTGCTCTAA − 3′. Each PCR amplified products was inserted into the pEcoli-Cterm6xHN vector (Clontech, Takara Bio USA) and then competent *E. coli* BL21(DE3) (Novagen, Merck Bioscience) cells were transformed with each recombinant plasmid and cultivated in LB broth (Amresco, USA) with addition of carbenicillin (Sigma-Aldrich). Expression of recombinant proteins were induced with 0.1 mM isopropyl- βD-thiogalactopyranoside (IPTG, Bioline, UK) at OD_600_ = 0.5–0.6, overnight 25 °C under shaking.

Recombinant proteins were purified by a combination of two methods: in first-round *E. coli* pellets (200 mg wet-weight per each clone) were lysed and purified according to the manufacturer’s instruction with HisTalon buffer set (Clontech, USA) and using Talon Metal affinity resin (Clontech, USA). Additional purification of protein eluates was achieved with ÄKTA pure protein purification system (GE Healthcare, USA), using HisTrap FF crude (GE Healthcare, Sweden) 1 mL columns according to the manufacturer’s instruction. Elution was done with a gradient of 20 mM to 200 mM imidazole in a buffer of 20 mM sodium phosphate pH 7.4 containing 0.5 M NaCl. Concentrations of dialyzed protein eluates were determined by Pierce bicinchoninic acid assay kit (BCA, Thermo Scientific, USA) [58].

### Human and animal sera

Possitive rabbit and BALB/c mice sera were prepared as described previously [27] with some small modifications. Briefly, 10^8^ live *Rickettsia akari* cells were intraperitoneally administered into rabbit in two consequent steps 28 days away. On the other hand, 30 μg of purified 44 kDa recombinant protein mixed with complete Freund’s adjuvant were intraperitoneally administered into the mice, followed by booster immunization with the same dose of protein combined with incomplete Freund’s adjuvant on the 28th-day after priming dose. Blood samples from the both experiments were collected at the 7th day after the booster by cardiac puncture under general anesthesia with sodium thiopental (for mice 50 mg/kg and 20 mg/kg for rabbit). Mice were sacrified by cervical dislocation and the rabbit was euthanized by intravenose overdose (100 mg/kg) of sodium thiopental (VUAB pharma, Czech republic). All animal experiments were conducted in the experimental animal facility at Biomedical Research Center of the Slovak Academy of Sciences (BMC, SAS) in accordance with the European Directive 2010/63/EU on the protection of animals used for scientific purposes and were approved by the ethical committee of BMC, SAS. The IgG titers of animal sera were determined by ELISA using the corresponding antigen.

The sera of rickettsia positive patients, diagnosed base on clinical signs and ELISA, were obtained from the National Reference Centre for Rickettsioses (established by Regional Authority of Public Health in Banska Bystrica, Slovakia). Before the experiments, sera were verified by IFA using a panel of rickettsial antigens, including SFG rickettsiae (*R. slovaca*, *R. conorii*, *R.helvetica, R. rickettsii*), *R. typhi* and *R. akari*. IFA test was considered positive when the antibody titers achieved a cutoff of 1/128 for IgG and 1/64 for IgM. A fourfold and greater increase above cutoff was recorded for three sera against *R. akari* antigen with positive clinical signs for rickettsialpox and for two sera against *R. slovaca*. Two sera from healthy blood donors were obtained from the serum collection of the Department of Rickettsiology, Biomedical Research Center of SAS in Bratislava.

### Serological analysis of *R. akari* antigen and recombinant proteins using western blot

*R. akari* proteins resolved by 2-DE were transferred (at 100 V/90 min) onto 0.45 μm PVDF membrane (Pall Life Sciences, USA) and blocked in 5% non-fat dried milk (Bio-Rad, USA) in PBS with 0.1% Tween-20 at 4 °C overnight. Membranes were incubated with a serum of rabbit infected with *R. akari* or serum from infected patients at 1:1000 dilutions. After incubation, the membranes were probed with horseradish peroxidase-conjugated polyclonal swine anti-rabbit (Dako Denmark A/S, 1:3000) or polyclonal rabbit anti-human IgG (Dako Denmark A/S, 1:3000) secondary antibody, respectively. Visualization was carried out with a method of enhanced chemiluminescence (ECL) [65].

In order to characterize the immunogenic properties of recombinant proteins, 16 μg of each purified proteins were resolved by 12% SDS PAGE gels and transferred to the PVDF membranes. Then, each membrane was sliced into 8 strips (containing approx. 2 μg of protein) and incubated separately with different sera from infected patients at 1:1000 dilutions. After incubation and repeated washings, the membrane strips were probed and visualized as described above.

### Preparation of “pre-absorbed” serum against rickettsial 44 kDa protein

The absorption procedure was described by Zhang et al. [66] with minor modifications. *E. coli* BL 21DE3 overnight cultures in LB broth with a density of 10^8^ cells/ml were centrifuged, washed twice and re-suspended in 100 μl of rickettsial 44 KDa protein mouse serum, incubated for 2 h at 37 °C and then overnight at 4 °C on a rotator. *E. coli* cells were centrifuged at 10000 x g for 30 min; the supernatant was collected and used for Immunofluorescence assay.

### Dual fluorescence staining of *R. akari* and 44 kDa uncharacterized protein (A8GP63)

Purified *R. akari* cells on a coverslip were fixed and permeabilized as described earlier [67]. Rickettsial cells were washed three times in PBS (pH 7.2) containing 2% bovine serum albumin (BSA; mixtures, PBSA) and then blocked with 5% BSA in PBS for 1 h at 37 °C. After washing, bacterial cells were incubated with a “pre-absorbed” mouse anti-serum against 44 kDa hypothetical protein (1:100) diluted in 2% PBSA for 1 h at 37 °C. After washing, cells were incubated with goat anti-mouse IgG secondary antibody conjugated with Alexa fluor 488 (Life Technologies, USA) diluted 1:1000 in PBS containing 2% PBSA. The cells were washed three times with PBSA and blocked again with 5% BSA in PBS for 1 h at 37 °C. *R. akari* cells were then stained by using polyclonal rabbit antiserum against live *R. akari* diluted 1:200 and a goat anti-rabbit IgG conjugated with Rhodamine (Life technologies, USA) diluted 1:2000. After five times washing with PBS, the coverslip was dried and mounted with Vectashield (Vector Laboratories) and viewed with fluorescence microscopy (model Eclipse Ni, Nikon Japan).

### Database use and in silico analyses

Prediction of membrane proteins was performed using four independent programs: signal peptide prediction of identified peptides was completed by the program SignalP-5.0 (http://www.cbs.dtu.dk/services/SignalP/) [68]. Predictions of the subcellular localization of identified proteins was performed by program PSORTb version 3.0.2 (https://www.psort.org/psortb/) [31] and SOSUI-GramN (http://harrier.nagahama-i-bio.ac.jp/sosui/sosuigramn/sosuigramn_submit.html) [32]. Prediction of beta-barrel outer membrane proteins was made by a hidden Markov model PRED-TMBB program (http://bioinformatics.biol.uoa.gr/PRED-TMBB/input.jsp) [33]. Homology of identified protein with other rickettsial proteins was compared using the Basic Local Alignment Search Tool (BLAST) (https://blast.ncbi.nlm.nih.gov/Blast.cgi) [69]. Protein classification into COGs functional classes was carried out using public database EggNOG v5.0 (http://eggnog5.embl.de/#/app/home) [70]. Analysis of the 44 kDa protein was submitted to the bcell program (http://tools.immuneepitope.org/bcell/) which predicts flexible linear B-cell epitopes. The sequence was submitted with a signal peptide and a threshold 0.35 [71].

## Supplementary information

**Additional file 1. **Identified *R. akari* proteins classified in the COGs functional category. * S - gel-free approach “Shotgun”; 2D - two dimensional electrophoresis; B - biotinylation; T- Triton X-114 phase partitioning.

**Additional file 2. ***R.akari* predicted surface-exposed proteins classified in the COGs functional category.

**Additional file 3.** Predicted B-cell epitopes of the 44 kDa uncharacterized protein (A8GP63). Threshold 0.35. The orientation of the peptide in the outer membrane is defined as out-faced to the external space, in-faced the internal lipid bilayer, and membrane-embedded in the outer membrane.

## Data Availability

The LC-MS/MS proteomics data have been deposited to the ProteomeXchange Consortium via the PRIDE (Perez-Riverol et al., 2019) partner repository with the dataset identifier PXD017762 and PXD018060 (https://www.ebi.ac.uk/pride/archive/login).

## Abbreviations

2-DE: Two-dimensional gel electrophoresis
ACN: Acetonitrile
AG: Ancestral group
ATP: Adenosine triphosphate
BSL3: Biosafety level 3
COG: Clusters of Orthologous Groups
ESI-LC-MS/MS: Electrospray Ionisation liquid chromatography coupled with tandem mass spectrometry
IPG: Immobilized pH gradient
kDa: Kilo Daltons
MW: Molecular weight
Omp: Outer membrane protein
p*I*: Isoelectric point
PVDF: Polyvinylidene fluoride
Sca: Surface cell antigen
SEP: Surface exposed protein
SFG: Spotted fever group
SPG: Sucrose phosphate glutamate
TG: Typhus group
TRG: Transitional group
