# Supplementary material for: Proteomic analysis of Rickettsia akari proposes a 44 kDa-OMP as a potential biomarker for Rickettsialpox diagnosis
Source: BMC Microbiol. 2020 Jul 8;20:200. doi: 10.1186/s12866-020-01877-6 (PMC7341715; doi:10.1186/s12866-020-01877-6)
Supplement: Supplementary file 3 — Additional file 3. Predicted B-cell epitopes of the 44 kDa uncharacterized protein (A8GP63). Threshold 0.35. The orientation of the peptide in the outer membrane is defined as out-faced to the external space, in-faced the internal lipid bilayer, and membrane-embedded in the outer membrane. [file 12866_2020_1877_MOESM3_ESM.doc]

**Additional file 3: Predicted B-cell epitopes of the 44 kDa uncharacterized protein (A8GP63)**

| **No.** | **Start** | **End** | **Peptide** | **Length** | **Orientation** |  |
| --- | --- | --- | --- | --- | --- | --- |
| 1 | 25 | 40 | SNIDDDMAIQENSKKS | 16 | out |  |
| 2 | 49 | 58 | KDMSSRASSD | 10 | in |  |
| 3 | 104 | 112 | EIGGHGARA | 9 | membrane |  |
| 4 | 124 | 130 | VGPFSGT | 7 | out |  |
| 5 | 149 | 154 | VDTGGV | 6 | in |  |
| 6 | 172 | 177 | NKINPT | 6 | out |  |
| 7 | 199 | 208 | NAKDKDGNDM | 10 | in |  |
| 8 | 305 | 309 | NQKFK | 5 | out |  |
| 9 | 346 | 355 | LKESCKQGGF | 10 | out |  |
| 10 | 373 | 378 | SGGYKT | 6 | out |  |

Threshold 0.35. The orientation of the peptide in the outer membrane is defined as out-faced to the external space; in-faced the internal lipid bilayer and membrane-embedded in the outer membrane.
